# Virosome Presents Multimodel Cancer Therapy without Viral Replication

**DOI:** 10.1155/2013/764706

**Published:** 2013-12-04

**Authors:** Kotaro Saga, Yasufumi Kaneda

**Affiliations:** Division of Gene Therapy Science, Graduate School of Medicine, Osaka University, 2-2 Yamada-oka, Suita, Osaka 565-0871, Japan

## Abstract

A virosome is an artificial envelope that includes viral surface proteins and lacks the ability to produce progeny virus. Virosomes are able to introduce an encapsulated macromolecule into the cytoplasm of cells using their viral envelope fusion ability. Moreover, virus-derived factors have an adjuvant effect for immune stimulation. Therefore, many virosomes have been utilized as drug delivery vectors and adjuvants for cancer therapy. This paper introduces the application of virosomes for cancer treatment. In Particular, we focus on virosomes derived from the influenza and Sendai viruses which have been widely used for cancer therapy. Influenza virosomes have been mainly applied as drug delivery vectors and adjuvants. By contrast, the Sendai virosomes have been mainly applied as anticancer immune activators and apoptosis inducers.

## 1. Introduction

Currently, general cancer therapies include surgery, chemotherapy, and radiation therapy, but all three have limitations. Applications of surgical and radiation therapy are limited to localized cancer. Chemotherapy is used for a wide range of cancers, including distant metastases, via the systemic administration of anti-cancer drugs; however, it also kills normal cells and induces severe side effects. Therefore, many groups are investigating ways to improve conventional treatments and to develop novel treatments for more effective cancer elimination with fewer side effects.

In recent years, much attention has been paid to cancer immunotherapy, which stimulates anti-cancer immunity, and several cancer immunotherapy systems (Provenge, Ipilimumab and anti-PD1 antibody) have been developed [1–5]. When anti-cancer immunity is systemically activated, it is expected that the primary cancer cells and distant metastases will be eliminated by immune cells. Various tumor-associated antigens (TAAs) have been identified [6–9], for example, HER2/nu, CEA, MAGE, and WT1. TAAs are expressed in cancer cells and are targeted by immune cells, especially cytotoxic T lymphocytes (CTL) [10–13]. Therefore, immunostimulation by TAAs can be applied to cancer immunotherapy. To activate anti-cancer immunity by TAAs, fragments of TAAs should be presented on antigen-presenting cells (APCs) by forming a complex with major histocompatibility complex class I (MHC-I) and II molecules [14]. Generally, cytoplasmic foreign proteins, such as viral proteins expressed in the cytoplasm during viral infection, complex with MHC-I and stimulate CD8^+^ T cells (CTLs) [14, 15]. However, endocytosed foreign proteins also complex with MHC-II and stimulate CD4^+^ T cells [14, 16]. Moreover, APCs have a cross-presentation system that presents endocytosed foreign proteins with MHC-I to activate CTLs [17]. Previous reports have shown that the administration of TAA alone does not induce an effective CTL response [18]. Therefore, it is believed that an endocytosed antigen is not sufficient for the activation of MHC-I-restricted CTLs, and, to activate an effective CTL response by TAAs, they should be introduced to the cytoplasm directly.

A new technology, gene therapy, has been developed and applied to cancer treatment. Various cancer gene therapy methods have been reported, such as adoptive immunotherapy using *ex vivo* gene transfer to immune cells [19], intratumoral injection of cytokine genes [20], suicide gene therapy using the herpes virus thymidine kinase gene [21], and intratumoral injection of the p53 gene [22]. To achieve high gene expression, viral vectors such as retrovirus and adenovirus vectors have been utilized. However, in general, cancer gene therapy has not had satisfactory therapeutic effects. Therefore, to enhance the cancer-cell-killing effect, viruses that replicate mainly in cancer cells have been used for treatment [23]. Various types of oncolytic viruses have been developed by isolating viruses with inherent tumor selectivity [24, 25] and by engineering recombinant viruses [26, 27]. Furthermore, the combination of an oncolytic virus and gene therapy has been applied for cancer treatment, such as vaccinia virus including the GM-CSF gene [28]. Although these oncolytic viral treatments exhibited a strong therapeutic effect, safety might be a problem because the virus with an intact genome still exists in noncancerous cells [29].

**Figure 1:**
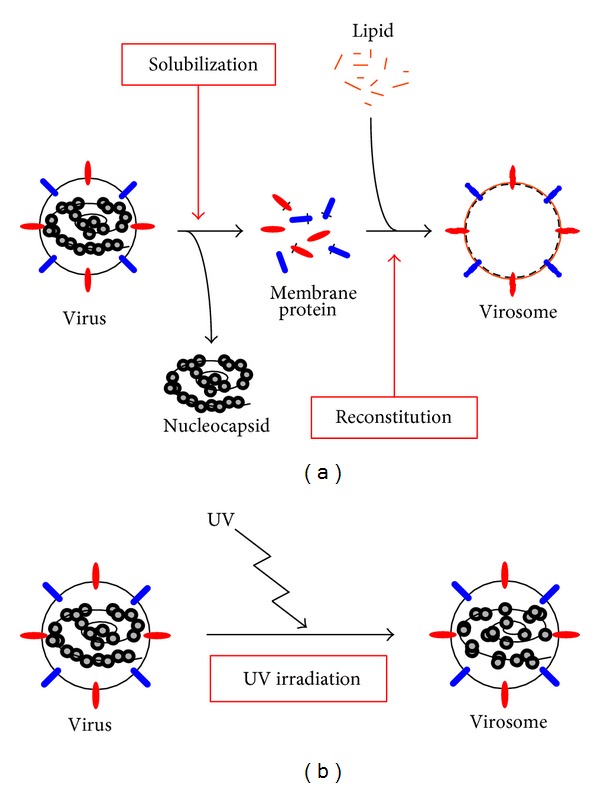
Concept of virosomes. (a) Reconstituted envelope containing viral envelope proteins. Viral membrane proteins are solubilized from viral particles, and artificial envelope is reconstituted with the viral proteins and exogenous lipids. (b) Viral envelope particles. Virus is inactivated with UV irradiation leading the fragmentation of viral genome.

An inactive virus that did not have the ability to amplify its progeny virus in host cells has also been used as a high-safety delivery vector for drugs and plasmids in cancer therapy. In particular, enveloped-virus-derived vectors have attracted attention because enveloped-vector-delivered molecules can escape endosomal degradation by direct introduction to the cytoplasm via membrane fusion [30]. A vector derived from an inactive enveloped virus is called a virosome, which is now an all-inclusive term for a reconstituted envelope that contains viral envelope proteins (Figure 1(a)) or viral envelope particles (Figure 1(b)) [31]. Several types of virosomes have been generated, for example, virosomes based on influenza virus [32], hepatitis B virus [33], human immunodeficiency virus [34], Newcastle disease virus [35], and Sendai virus [36, 37]. In many studies, virosomes have been used as vectors for drug delivery, with the inclusion of various therapeutic molecules, such as DNA, RNA, proteins, and drugs [38, 39]. Moreover, virosomes function as adjuvants to induce the activation of the immune system [40]; therefore, many groups are studying virosomes as tools for cancer therapy.

In this review, we introduce the previous research on virosomes, especially virosomes derived from the influenza (influenza virosome) and Sendai viruses (Sendai virosome) for the use in cancer therapy. The influenza virosome has been applied mainly as a delivery vector for TAAs and TAA-expressing plasmids. Sendai virosomes have been used as anti-cancer immune activators and apoptosis inducers.

## 2. Influenza Virosomes

Influenza virus is an Orthomyxovirus that has a nucleocapsid with a segmented single-stranded RNA genome and is covered with a viral envelope [41, 42]. Two types of membrane proteins, hemagglutinin (HA) and neuraminidase (NA), are present on the surface of the envelope. HA binds to sialic acid, which is its receptor, on the surface of host cells and is used for the adhesion of viral particles [43]. HA is responsible for membrane fusion of the viral envelope with the host cell membrane [44]. However, HA does not induce membrane fusion in neutral conditions, and it acquires its fusion activity through conformational change in acidic conditions [45, 46]. Viral particles are taken into the endosomes of host cells by endocytosis after HN-receptor binding, thereby exposing the particles to acidic conditions. Next, membrane fusion of the viral envelope with the endosomal membrane is induced by the conformational change of HA, and the viral genome is induced into the cytoplasm of host cells.

An influenza virosome is an artificial liposome that includes influenza membrane proteins [31] and is prepared by reconstituting influenza virus surface proteins and phospholipids [47]. The influenza viral envelope is first collapsed to phospholipids by the treatment with detergent, and the nucleocapsid is eliminated from the mixture. Then, the influenza virosome, including surface proteins and virus-derived phospholipids, is reconstituted from the mixture. An influenza virosome maintains its membrane fusion ability because it has HA on its surface [48]. Therefore, it works as a delivery vector to introduce macromolecules into the cytoplasm by including them in the virosome [38, 49]. Influenza virosomes have powerful immunogenicity. Vaccination with influenza virosomes induces protective levels of influenza-specific antibodies [50], and an influenza virosome is already licensed as an influenza vaccine [51]. Influenza virosomes also exhibit an adjuvant effect when they are coadministered with other antigens [52–54]; therefore, many groups have studied the application of influenza virosomes in the activation of antitumor immunity.

### 2.1. CTL Activation by Plasmid DNA Encapsulation in Influenza Virosomes

Correale et al. reported that TAA-specific CTLs were induced by the administration of an influenza virosome containing TAA plasmids in mice [55]. In this study, a plasmid expressing parathyroid hormone-related peptide (PTH-rP), which is a TAA expressed in prostate and spinocellular lung carcinomas, was included in an influenza virosome, which was administered intranasally. As a result, PTH-rP-specific CTL activity was significantly induced in mice, and this activity was also shown in human PBMCs activated by human DCs treated with the PTH-rP virosome. In addition, Cusi et al. demonstrated that TAA-specific CTLs were enhanced by the stimulation with an influenza virosome containing a CD40L-expressing plasmid [56]. CD40L binds to CD40 on APCs and upregulates the expression of its costimulatory molecules, B7.1 and B7.2, in the cells, which are important factors for the activation and amplification of naïve T cells [57, 58]. In this study, plasmids expressing carcinoembryonic antigen (CEA), which is a marker of colon cancer, and CD40L were encapsulated in influenza virosomes, and these virosomes were administered intranasally. Coadministration of CEA- and CD40L-virosomes resulted in a CEA-specific CTL response that was stronger than that in the CEA-virosome alone, by upregulating B7.1 and B7.2 expression on APCs.

### 2.2. CTL Activation by Peptide Encapsulation in Influenza Virosomes

Antigen presentation of TAAs by APCs is important for the activation of anti-cancer immunity. To activate CTLs, TAAs should be presented with MHC-I, which complexes with cytoplasmic antigens. Therefore, TAAs should be introduced to the cytoplasm for the effective activation of CTLs. Bungener et al. demonstrated influenza virosome-mediated OVA delivery to DCs [59] and that the delivery leads to OVA presentation on MHC-I and -II. Fusion-inactive virosomes presented OVA on MHC-II but not on -I. Therefore, it is suggested that influenza virosomes introduce encapsulated TAAs to the cytoplasm through membrane fusion and that TAA introduction is needed for the presentation of TAAs on MHC-I. Angel et al. reported influenza virosome-mediated delivery of TAAs to DCs [60]. The authors encapsulated the Melan-A peptide, which is a TAA from melanoma, in an influenza virosome and introduced the Melan-A peptide into plasmacytoid DCs (PDCs). Melan-A-containing, virosome-treated PDCs activated CD8 T cells more effectively than did free Melan-A peptide-pulsed PDCs. In addition, Correale et al. reported that PTH-rP-derived peptide (PTR)-4-encapsulated influenza virosomes significantly suppressed tumor growth [61]. In this study, PTR-4/virosome treatment effectively activated CTL activity, and the treatment inhibited the angiogenesis of tumors. The findings therefore suggest a new function of influenza virosomes in cancer therapy.

### 2.3. Modification of the Influenza Virus

To make influenza virosome-mediated cancer therapy more effective, modifications of the influenza virosome have been attempted. HA has an important function in influenza virosome-mediated delivery and immunostimulation. However, the HA receptor is ubiquitously expressed on nearly all cells. Therefore, the influenza virosome does not have affinity for specific cells. Mastrobattista et al. generated an influenza virosome that could target ovarian carcinoma (OVCAR-3) *in vitro* [62]. They coated influenza virosomes with polyethylene glycol (PEG) to inhibit HA-mediated binding, and then Fab' fragments of antiepithelial glycoprotein-2 (EGP-2) antibody (323/A3) were conjugated to the PEG on the virosomes. 323/A3-PEG-coated influenza virosomes exhibited low HA-mediated binding to sialic acid because of the PEG coating and gained specific binding for EGP-2-expressing ovarian cancer cells by 323/A3 conjugation. As a result, although the binding function of HA was depleted, the 323/A3-PEG virosomes were able to fuse with OVCAR-3 membranes. Because HA induced membrane fusion without binding to its receptor [63], it is thought that the 323/A3-PEG virosomes maintained their membrane fusion ability. Waelti et al. used the same strategy to demonstrate targeted delivery of doxorubicin (Doxo) to HER-2/neu-overexpressing breast cancer cells *in vivo* [64]. In this study, influenza virosomes were coated with anti-Neu mAb Fab' (7.16.4)-conjugated PEG (7.16.4/PEG), and Doxo was encapsulated in the 7.16.4/PEG-virosomes. Intravenous administration of Doxo-containing 7.16.4/PEG-virosomes significantly inhibited subcutaneous Neu+, but not Neu-, breast cancer. Jamali et al. recently reported the enhancement of the efficacy of influenza virosome-mediated delivery *in vitro* by reconstituting the virosome with cationic lipids [65].

As described above, influenza virosomes are useful for the cancer therapy. Recently, phase I clinical trial of influenza virosomes was carried out for the patients with metastatic breast cancer (MBC) [66]. In this trial, MBC patients were intramuscularly administrated influenza virosomes including three individual peptides of the extracellular domain of Her-2/neu protein. The trial tested the safety and Her-2/neu-specific immune responses. As a result, specific antibodies against naïve Her-2/neu protein were detected in serum. IL-2 production was significantly increased and Treg population was significantly decreased in PBMC. Although local erythema at the infection site has appeared in four patients, other serious side effects were not detected. Therefore, there is a possibility that influenza virosomes are used for future cancer therapy.

## 3. Sendai Virosomes

Sendai virus (hemagglutinating virus of Japan; HVJ) is a paramyxovirus that has a nucleocapsid with a single-stranded RNA genome and is covered with a viral envelope [67]. Two types of glycoproteins, hemagglutinin-neuraminidase (HN) and fusion protein (F), are present on the surface of the viral envelope [68]. HN enables the viral particle to adhere to the host-cell surface by binding to sialic acid [69], and then F induces membrane fusion of the viral envelope with the host-cell membrane [70]. F fuses these membranes under neutral conditions [71]; therefore, HVJ particles do not require uptake into the endosome for membrane fusion.

Previously, HVJ-liposomes were generated via reconstitution from HVJ surface proteins and phospholipids, similar to the influenza virosome [36]. Because HVJ-liposomes have membrane fusion ability, they have been used as a vector for DNA delivery [72]. However, because the membrane fusion efficiency of HVJ-liposomes is not high (approximately 2% of native HVJ) [73], an increase in the fusion activity of the vector is needed.

Kaneda et al. generated a new type of Sendai virosomes called HVJ-envelope (HVJ-E) [37]. HVJ-E is an inactivated HVJ particle that has been irradiated by UV light. The viral RNA genome is cleaved into many fragments; therefore, HVJ-E does not have the ability to produce progeny virus in infected cells. However, HVJ-E maintains its membrane fusion ability, which is dramatically higher than that of HVJ-liposomes [37]. HVJ-E has been used as a vector for plasmid DNA delivery to various cells and tissues [74–76]. In addition, plasmid DNA, anti-cancer drugs, and siRNAs have been delivered by HVJ-E, and there have been reports of cancer therapy using HVJ-E-mediated drug delivery [77, 78].

Cancer suppression by viral infection has also been reported [79]. Since that study, various viruses have been used for cancer therapy, and, in particular, the development of oncolytic viruses has attracted attention [80–83]. Oncolytic viruses function by inducing the lysis of cancer cells by infection [84]. Because the oncolytic activity is decreased by UV irradiation, it has been suggested that the viral amplification in cancer cells is responsible for oncolysis [85]. However, it is also possible that the virus's components contribute to the suppression of cancer. Recently, it was indicated that HVJ-E itself has an inhibitory effect against cancer growth [86, 87], and it was revealed that the viral components, in the absence of viral amplification, contribute to the anti-cancer effects. Since then, the HVJ-E-mediated anti-cancer effect has been studied.

### 3.1. HVJ-E for the Activation of Anticancer Immunity

**Figure 2:**
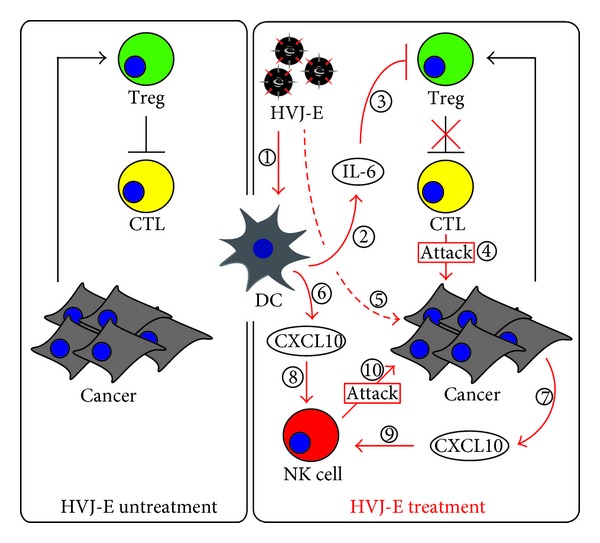
HVJ-E-mediated stimulation of anticancer immunity. Various modes of stimulations of the immune system that are induced by HVJ-E treatment. Black lines indicate the original reactions in cancer. Red lines indicate the reactions induced by HVJ-E treatment. *CTL Activation*. 1: HVJ-E stimulates dendritic cells (DCs). 2: DC secretes IL-6. 3: IL-6 suppresses regulatory T cell (Treg) function, which inhibits cytotoxic T cell (CTL) activity. 4: CTLs attack cancer cells. *NK Cell Activation*. 1, 5: HVJ-E stimulates DCs and cancer cells. 6, 7: DCs and cancer cells secrete CXCL10. 8, 9: CXCL10 attracts natural killer (NK) cells to cancer cells. 10: NK cells effectively attack the cancer cells.

Kurooka and Kaneda demonstrated that the intratumoral administration of HVJ-E dramatically eradicated intradermal cancer (Figure 2) [86]. They found that HVJ-E stimulated DCs to release various types of cytokines, such as interferon (IFN)-*α*, and -*β*, tumor-necrosis factor (TNF)-*α* and interleukin (IL)-6, and that IL-6 inhibited the proliferation of regulatory T cells (Tregs). Tregs negatively control effector T cells [88, 89] and interfere with the activation of anti-cancer immunity [90]. Therefore, HVJ-E-mediated eradication of cancer results from the activation of anti-cancer immunity by IL-6-mediated suppression of Tregs. It is known that RNA viruses stimulate DCs via the recognition of the viral RNA genome by Toll-like receptor (TLR)-7 and -8 and Rig-I [91–93]. However, Suzuki et al. showed that the sugar chain of the F protein is important for HVJ-E-mediated, DC activation of IL-6 secretion [94]. Therefore, they suggested that DCs possess an unknown receptor for F that is involved in maturation.

In addition, HVJ-E suppressed tumor growth in the intradermal renal carcinoma SCID mouse model, in spite of their deficient T and B cells [95], which suggests that HVJ-E undergoes another stimulation mechanism that activates anti-cancer immunity. Fujihara et al. indicated that the anti-cancer activity of NK cells was led by the intratumoral administration of HVJ-E into the intradermal renal carcinoma SCID mouse model (Figure 1) [95]. In addition, HVJ-E directly stimulated cancer cells and induced their secretion of CXCL10. CXCL10 is a chemokine for monocytes/macrophages, T cells, NK cells, and DCs; therefore, it is suggested that active NK cells were attracted to the tumor by CXCL10.

Taken together, these reports demonstrate that HVJ-E is a powerful activator of anti-cancer immunity.

### 3.2. Direct Cancer Killing Activity of HVJ-E

**Figure 3:**
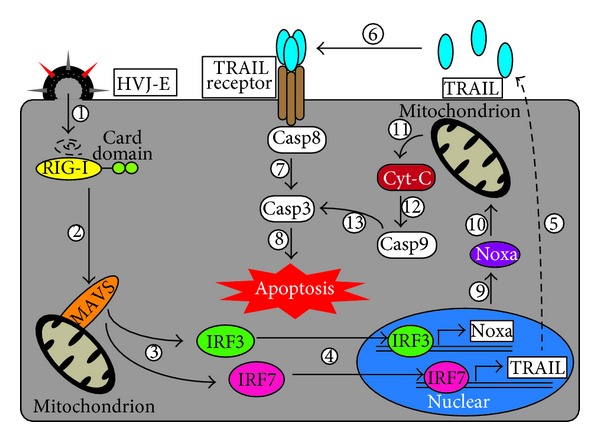
HVJ-E-mediated apoptosis pathway in cancer cells. HVJ-E-mediated signal transduction in cancer cells. 1: RNA fragments derived from the HVJ genome are introduced into the cytoplasm by membrane fusion, and RIG-I recognizes these RNAs. 2: RIG-I conveys the signal to the mitochondrial antiviral signaling adaptor (MAVS). 3: MAVS activates IRF-7 and -3. 4: activated IRF-7 and -3 localize to the nucleus. 5, 9: IRF-7 and -3 induce the expression of TRAIL and Noxa. 6: expressed TRAILs are recognized by the TRAIL receptor, and TRAIL receptors activate caspase-8 (Casp-8). 7: activated Casp-8 activates Casp-3. 8: activated Casp-3 induces apoptosis. 10, 11: Noxa induces the secretion of cytochrome-C (Cyt-C) into the cytoplasm from the mitochondria. 12: Cyt-C activates Casp-9. 13: Casp-9 activates Casp-3.

Recently, it was reported that HVJ-E has a direct killing effect against cancer cells (Figure 3). Kawaguchi et al. showed that the viability of two castration-resistant human prostate cancer cell lines (PC3 and DU145) was remarkably decreased by the treatment with HVJ-E *in vitro *[96]. HVJ-E-treated PC3 cells exhibited some apoptotic phenotypes, namely, increases in the number of TUNEL-stained cells and in the expression levels of caspase-3 and caspase-8. However, HVJ-E-mediated inhibition of cell viability was not observed in normal prostate epithelium (PNT2), suggesting that HVJ-E-mediated apoptosis is specifically induced in cancer cells. HVJ-E contains many fragments of its RNA genome, and these RNA fragments are introduced to the cytoplasm by the fusion of HVJ-E and the cell membrane. Matsushima-Miyagi et al. revealed that the viability of prostate cancer cells (PC3 and DU145), but not normal prostate epithelium (PNT1 and PNT2), was significantly decreased by viral RNA introduction (Figure 2) [87]. The RNA fragments were recognized by RIG-I in the cytoplasm, and the signal was transduced to MAVS [97]. HVJ-E-mediated cell growth inhibition of PC3 was suppressed by the knockdown of RIG-I and MAVS, indicating that the RIG-I/MAVS signaling pathway is important for this process. Moreover, HVJ-E treatment induced the expression of TRAIL and Noxa (known as apoptosis inducers [98, 99]) in PC3 and DU145 cells, but not in PNT2 cells, via RIG-I/MAVS signaling. The fact that the knockdown of TRAIL and Noxa suppressed the HVJ-E sensitivity of PC3 and DU145, respectively, indicates that these apoptosis inducers are responsible for HVJ-E-induced cancer cell apoptosis. Furthermore, the knockdown of IRF7 and 3—transcription factors of TRAIL and Noxa, respectively [100, 101]—also suppressed the HVJ-E sensitivity of prostate cancer cells, suggesting that RIG-I/MAVS signaling regulates the expression of TRAIL and Noxa via IRF7 and 3 in cancer cells. Matsushima-Miyagi et al. [87] elucidates the mechanism of HVJ-E-induced cancer cell apoptosis. However, it is still unknown why the expression of these apoptosis inducers is induced in cancer cells by HVJ-E stimulation.

### 3.3. Combination Therapy with HVJ-E and Modification of HVJ-E

In attempts to enhance the strength and decrease the side effects of HVJ-E-mediated antitumor treatment, various combination therapies that include HVJ-E and modifications of HVJ-E have been used. Eg5 is an important factor in the early stages of mitosis [102] and its inhibition leads to mitotic arrest and results in apoptosis [103]. Matsuda et al. demonstrated that HVJ-E-mediated apoptosis in human glioblastoma cell lines (A-172, T98G and U-118MG) was effectively enhanced by the encapsulation of siRNAs against Eg5 in HVJ-E *in vitro *and* in vivo* [104]. The authors also observed that HVJ-E-mediated anti-cancer immunity was enhanced by the encapsulation of the IL-2 plasmid and that the astrocytoma cell line (RSV-M) was effectively eradicated when using this method* in vivo* [105].

HVJ-E adheres to the cell surface via HN binding to sialic acid (e.g., GD1a and SPG) [106]. Therefore, cancer cells with mild expression of these sialic acids exhibit low sensitivity to HVJ-E-mediated apoptosis because of their weak affinity for HVJ-E. To induce HVJ-E-mediated apoptosis in less sensitive cancer cells, Nomura et al. used the combination therapy of HVJ-E and 13-cis retinoic acid (13cRA) against human neuroblastoma cells (NB1), which are less sensitive to HVJ-E [107]. NB1 cells barely express GD1a and SPG and exhibit low sensitivity to HVJ-E-mediated apoptosis. 13cRA treatment induced the expression of GD1a in NB1 cells, and the HVJ-E sensitivity of NB1 cells was increased* in vitro*. Moreover, NB1 tumor volume in mice was significantly decreased and their survival rate was increased by the combination of HVJ-E and 13cRA *in vivo*.

Improvements to HVJ-E were made to enhance its performance. Sialic acids, such as GD1a and SPG, to which HN bind, are ubiquitously expressed in nearly all cells, and they are highly expressed in red blood cells. Therefore, HVJ-E does not have an affinity for a specific cell type, and it induces hemagglutination by intravenous administration. For the systemic administration of HVJ-E to treat cancer effectively, it must have high affinity for cancer cells and low affinity for sialic acids. Transferrin (Tf) is a protein in blood plasma that is responsible for ferric ion delivery, and the Tf receptor is highly expressed in various cancer cells. Shimbo et al. generated a cancer-targeting HVJ-E using Tf [108]. The HN on HVJ-E was depleted by siRNA [109], and Tf was presented on the surface of HVJ-E via the expression of a Tf/F recombinant fusion protein on HVJ-E. Tf-presented HVJ-E (Tf-HVJ-E) exhibited affinity for the human uterocervical cancer cell (Hela) line, which expressed the Tf receptor, and Tf-HVJ-E accumulated at tumor masses in mice after their systemic administration.

In addition, HVJ-E-mediated antitumor immunity was enhanced by HVJ-E modification. HVJ-E activates anti-cancer immunity; however, HVJ-E does not directly induce IFN-*γ* secretion. IFN-*γ* is an important factor for various anti-cancer activities [110], and IL-12 is a robust inducer of IFN-*γ* from immune cells [111, 112]. Saga et al. revealed that HVJ-E dramatically enhanced IL-12 activity for IFN-*γ* secretion from splenocytes; however, HVJ-E alone did not induce IFN-*γ* secretion [113]. The authors generated IL-12-conjugated- and HN-depleted HVJ-E (IL-12-HVJ-E) to enhance HVJ-E-mediated anti-cancer immunity. IL-12-HVJ-E induced secretion of IFN-*γ* from splenocytes *in vitro*. In addition, upon intratumoral injection, scIL12-HVJ-E activated antitumor immunity against mouse malignant melanomas (F10 melanoma) and suppressed tumor growth more effectively than the wild-type (wt) HVJ-E. Furthermore, upon intravenous injection, IL-12-HVJ-E, but not wt-HVJ-E, was especially localized to the lungs, where it induced IFN-*γ* expression and reduced the lung metastatic foci of F10 melanomas.

As described above, HVJ-E has the ability to induce anti-cancer effects in several types of cancers. Now, clinical trials of HVJ-E are ongoing to test its safety and anti-cancer immunity against melanoma and prostate cancer. Moreover, there is a possibility that the combination therapy of HVJ-E and other immune therapies, such as CTLA-4 antibody, exhibits a more effective activation of antitumor immunity, and it will be performed in the near future.

## 4. Conclusion

We have documented the utility of virosomes for cancer treatment. However, we believe that no omnipotent therapeutic technologies are currently available to completely eradicate various types of cancers. Cancers are heterogeneous and can transform themselves to be resistant to the treatment that they have received and to escape from the environment of cancer treatment [114]. In this scientific research field, it is absolutely necessary to identify the genes that direct tumorigenesis. However, in the clinical field, it is very important to prepare cancer treatments using a variety of therapeutic principles. Clinicians should provide cancer patients with the appropriate therapeutic tools according to the patient's condition. Thus, from a practical standpoint, virosome-mediated cancer therapy may have an important role in cancer treatment.
